# Molecular mechanism underlying the effect of maleic hydrazide treatment on starch accumulation in *S. polyrrhiza* 7498 fronds

**DOI:** 10.1186/s13068-021-01932-y

**Published:** 2021-04-19

**Authors:** Yerong Zhu, Xiaoxue Li, Xuan Gao, Jiqi Sun, Xiaoyuan Ji, Guodong Feng, Guangshuang Shen, Beibei Xiang, Yong Wang

**Affiliations:** 1 College of Life Science, Nankai University, Weijin Road 94, Tianjin, 300071 China; 2 School of Chinese Material Medica, Tianjin University of Traditional Chinese Medicine, Poyang Lake Road 10, Tianjin, 301617 China

**Keywords:** Starch accumulation, Maleic hydrazide, Carbon fixation, Molecular mechanism

## Abstract

**Background:**

Duckweed is considered a promising feedstock for bioethanol production due to its high biomass and starch production. The starch content can be promoted by plant growth regulators after the vegetative reproduction being inhibited. Maleic hydrazide (MH) has been reported to inhibit plant growth, meantime to increase biomass and starch content in some plants. However, the molecular explanation on the mechanism of MH action is still unclear.

**Results:**

To know the effect and action mode of MH on the growth and starch accumulation in *Spirodela polyrrhiza *7498, the plants were treated with different concentrations of MH. Our results showed a substantial inhibition of the growth in both fronds and roots, and increase in starch contents of plants after MH treatment. And with 75 µg/mL MH treatment and on the 8th day of the experiment, starch content was the highest, about 40 mg/g fresh weight, which is about 20-fold higher than the control. The I_2_-KI staining and TEM results confirmed that 75 µg/mL MH-treated fronds possessed more starch and big starch granules than that of the control. No significant difference for both in the photosynthetic pigment content and the chlorophyll fluorescence parameters of PII was found. Differentially expressed transcripts were analyzed in *S. polyrrhiza* 7498 after 75 µg/mL MH treatment. The results showed that the expression of some genes related to auxin response reaction was down-regulated; while, expression of some genes involved in carbon fixation, C4 pathway of photosynthesis, starch biosynthesis and ABA signal transduction pathway was up-regulated.

**Conclusion:**

The results provide novel insights into the underlying mechanisms of growth inhibition and starch accumulation by MH treatment, and provide a selective way for the improvement of starch production in duckweed.

**Supplementary Information:**

The online version contains supplementary material available at 10.1186/s13068-021-01932-y.

## Background

Duckweeds (*Lemnaceae*) are among the known smallest flowering plants, which are aquatic, propagate mainly via asexual reproduction, and most importantly grow more rapidly than other higher plants [1–3]. These tiny plants usually have high starch content and, therefore, have been developing into very promising bioenergy resources [3, 4]. It has been reported that the starch content of duckweed can be elevated by subjecting the plants to nutrient starvation or treating them with plant growth regulators, which results in inhibition or delay of asexual propagation [5–10].

Maleic hydrazide (MH) is one of the plant growth regulators and has been shown to inhibit plant growth without causing obvious morphological abnormalities [11, 12]. Therefore, it has been widely used as a temporary growth inhibitor in many plants. For example, it has been reported to delay the development of buds, so that vegetables can last longer in storage and arrive fresher to the consumer [13, 14] and was also used for control of axillary growth (suckers) in tobacco production, to alter cured-leaf quality, and to increase yield [15].

Despite many studies of MH application, the mode of its action is less investigated. In this paper, we explored the effect and mechanism of MH on inhibiting growth in plants of *Spirodela polyrrhiza* 7498, the first duckweed species used for genome sequencing, as an appropriate concentration can promote starch content, which is very feasible in the application of bioenergy production of duckweed plants [3, 16, 17, 30].

## Results

### Effect of MH on vegetative growth

**Fig. 1:**
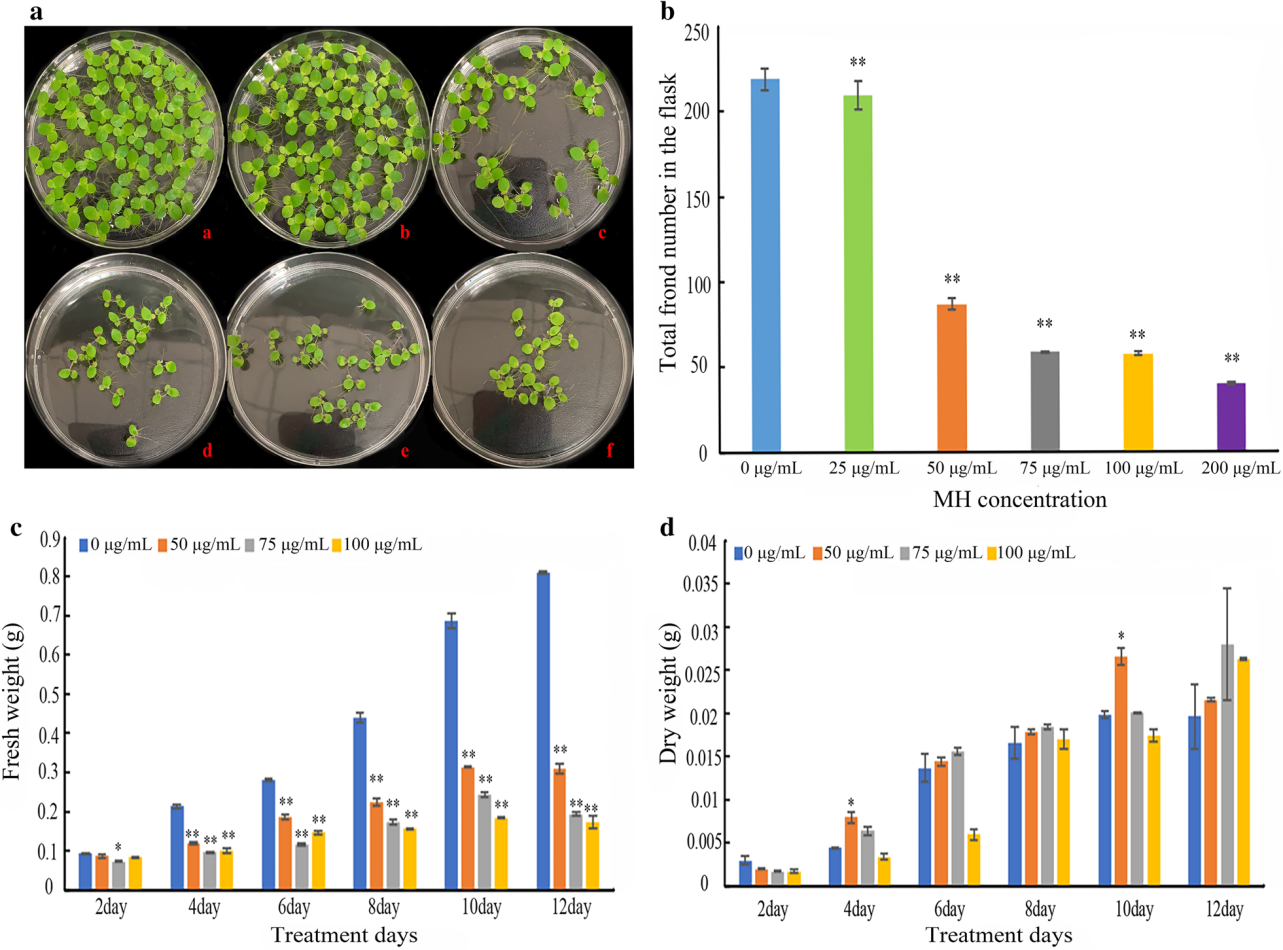
Effect of MH on the growth of *S. polyrrhiza* 7498. Six groups of duckweed plants (total 24 fronds) were inoculated into the flasks with different medium, respectively. On the 8th day of experiment, photos (**a**) were taken, and frond numbers (**b**) were accounted. Fresh and dry weight (**c**, **d**) were measured every 2 days. In **a** a to f show plants incubated in 0 µg/mL, 25 µg/mL, 50 µg/mL, 75 µg/mL, 100 µg/mL and 200 µg/mL MH, respectively. * indicates statistically significance on the level of P < 0.05; and ** indicates statistically significance on the level of *P* < 0.01, in comparison with the control (0 µg/mL). Each point in **b**–**d** represents mean ± SE of 3 biological replicates

Plants were inoculated into flasks with fresh medium containing different concentration of MH, respectively, and cultivated under conditions described in the “Material and methods” part. On the 8th day of experiment, the effect of MH on plant growth was observed. While the control plants, cultivated on the medium without MH, grew very quickly, the growth of plants on medium containing MH was inhibited in a concentration-dependent manner. As shown in Fig. 1, both total frond number and fresh weight were decreased by MH treatment in the concentration range from 25 to 200 µg/mL. MH-treated plants also had fewer and shorter roots (rhizoid) than the controlled plants (Additional file 1: Figure S1). Vegetative reproduction was almost completely inhibited at 75 µg/mL MH and higher concentrations. However, dry weight was not significantly affected even by 100 µg/mL MH (Fig. 1d).

### Effect of MH on starch accumulation

**Fig. 2:**
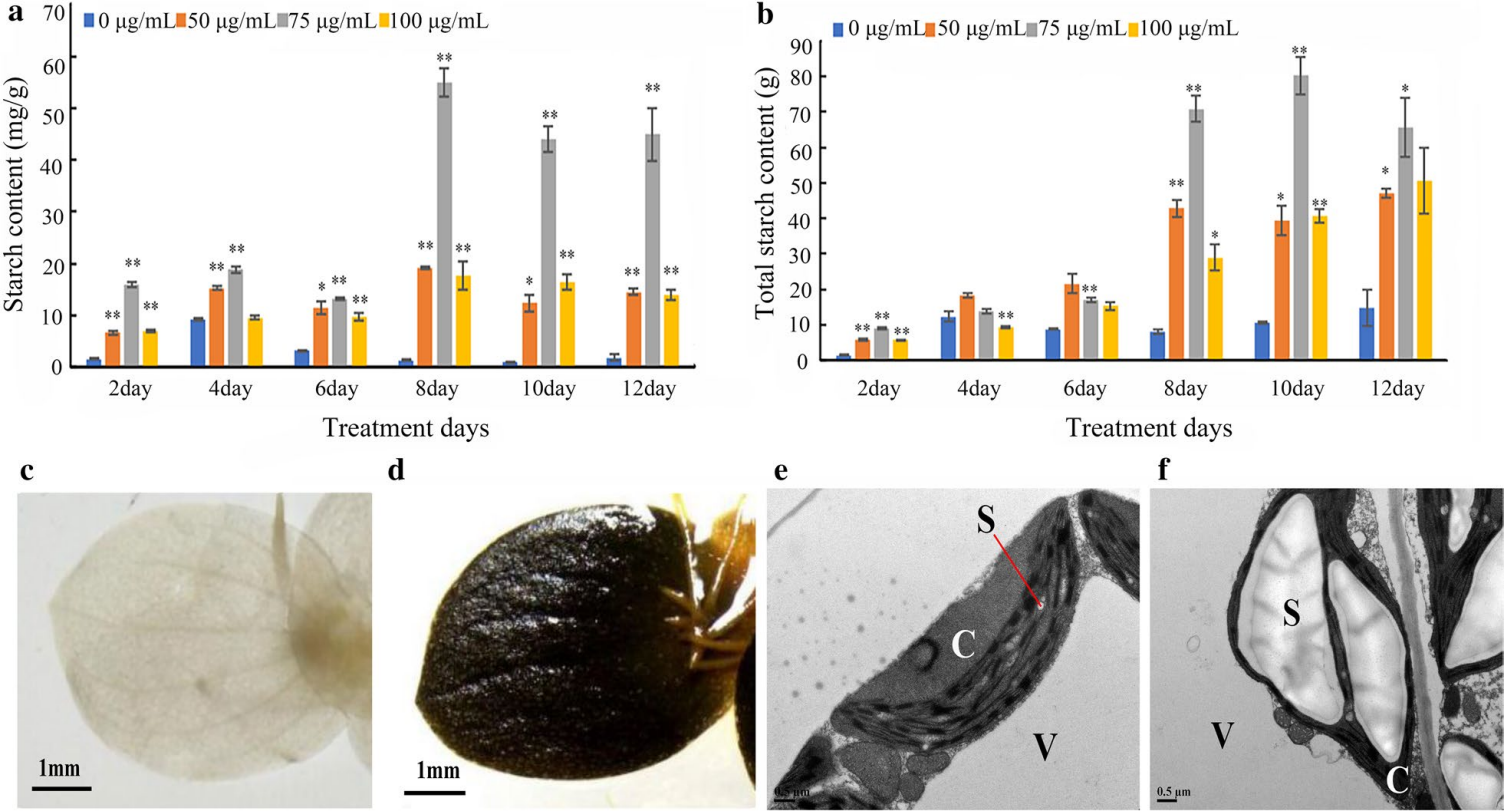
Starch accumulation in *S. polyrrhiza* 7498 plants after MH treatment. Starch content was calculated based on fresh weight (**a**); or each flask (**b**). Plants cultivated on the medium without (**c** and **e**) or with 75 µg/mL MH (**d** and **f**) were collected on the 8th day of the experiment and stained with I_2_-KI (**c** and **d**) or subjected to starch granule observation (**e** and **f**) with TEM. C, V and S represents chloroplast, vacuole and starch granule, respectively. * indicates statistically significance on the level of *P* < 0.05; and ** indicates statistically significance on the level of *P* < 0.01. Each point in A and B represents mean ± SE of 3 biological replicates

We measured the starch content of *S. polyrrhiza* 7498 during a cultivation period of 12 days. While the starch content in the plants grown on medium without MH was maintained in a similarly low level, dramatical increases of starch content were found in plants grown on medium with all concentrations of MH (Fig. 2a, b). The highest starch content was demonstrated in plants grown on medium with 75 µg/mL MH at the 8th day of the experiment (Fig. 2a, b). In these plants, the starch content was about 60 mg per gram fresh weight, which was about 20-fold higher than that of the control, and the total starch yield was about sevenfold higher than that of the control. This dramatic increase in starch content was further confirmed by staining the fronds with I_2_-KI (Fig. 2c, d). Then, Transmission Electron Microscope (TEM) was used to investigate the starch granules in mesophyll cells. It was found that there were more big starch granules in the chloroplasts of MH-treated fronds than that of the control (Fig. 2e, f).

### Effect of MH on parameters related to light reactions

**Fig. 3:**
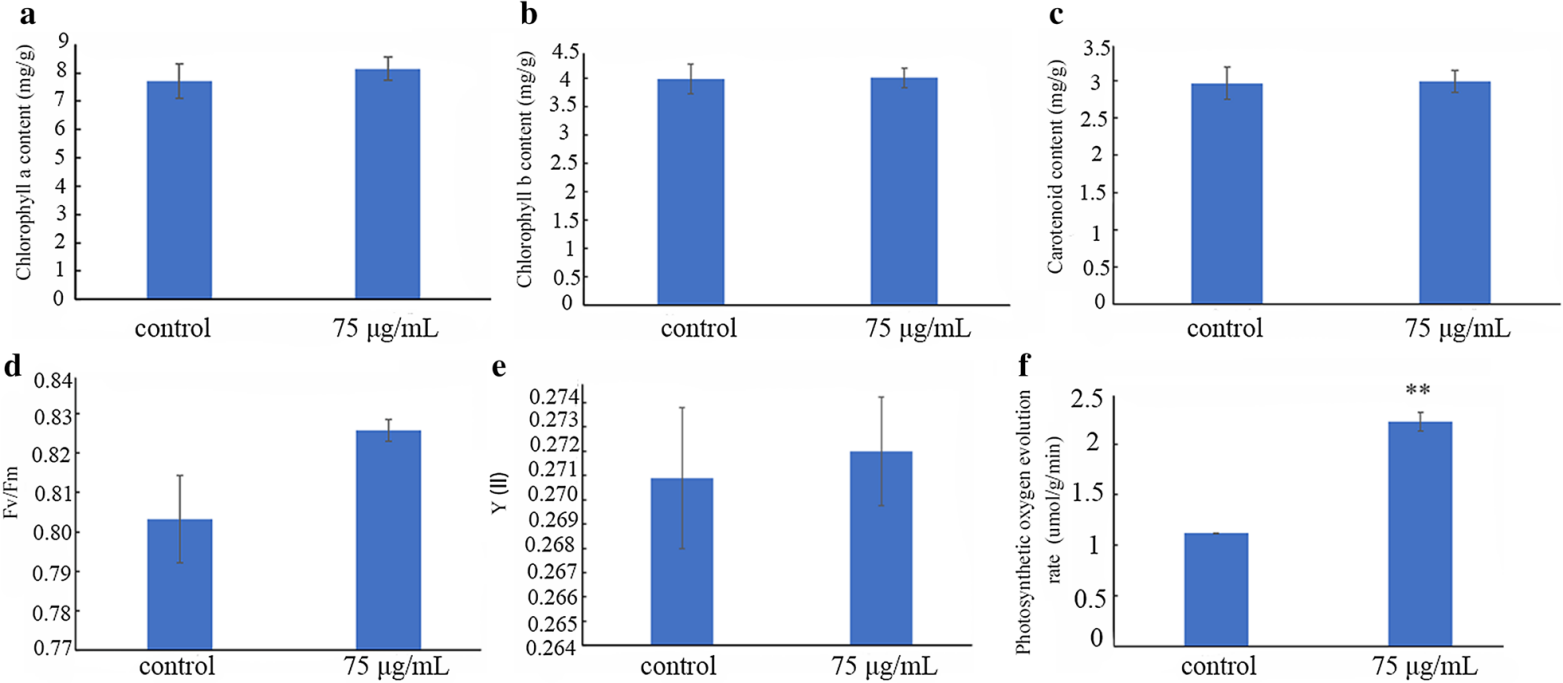
Comparison of photosynthetic parameters in *S. polyrrhiza* 7498 plants. Photosynthetic parameters were determined on the 8th day of experiment with 75 µg/mL MH. These include pigment content (**a**–**c**), fluorescence parameters of PSII (**d** and **e**) and rate of photosynthetic oxygen evolution (**f**). ** in **f** indicates statistically significance (*P* < 0.01). Each point in the figure represents mean ± SE of 3 biological replicates

To figure out the reasons for starch accumulation after MH treatment, we investigated parameters related to light reactions of photosynthesis. The results showed that there was no significant difference in photosynthetic pigment contents, but the chlorophyll fluorescence parameters PSII and Fv/Fm were increased in fronds treated by 75 µg/mL MH (Fig. 3a–e). Additionally, the rate of photosynthetic oxygen evolution from the MH-treatment fronds was also significantly higher than that of the control (Fig. 3f).

### Effect of MH on morphology, structure and stomatal opening of fronds

**Fig. 4:**
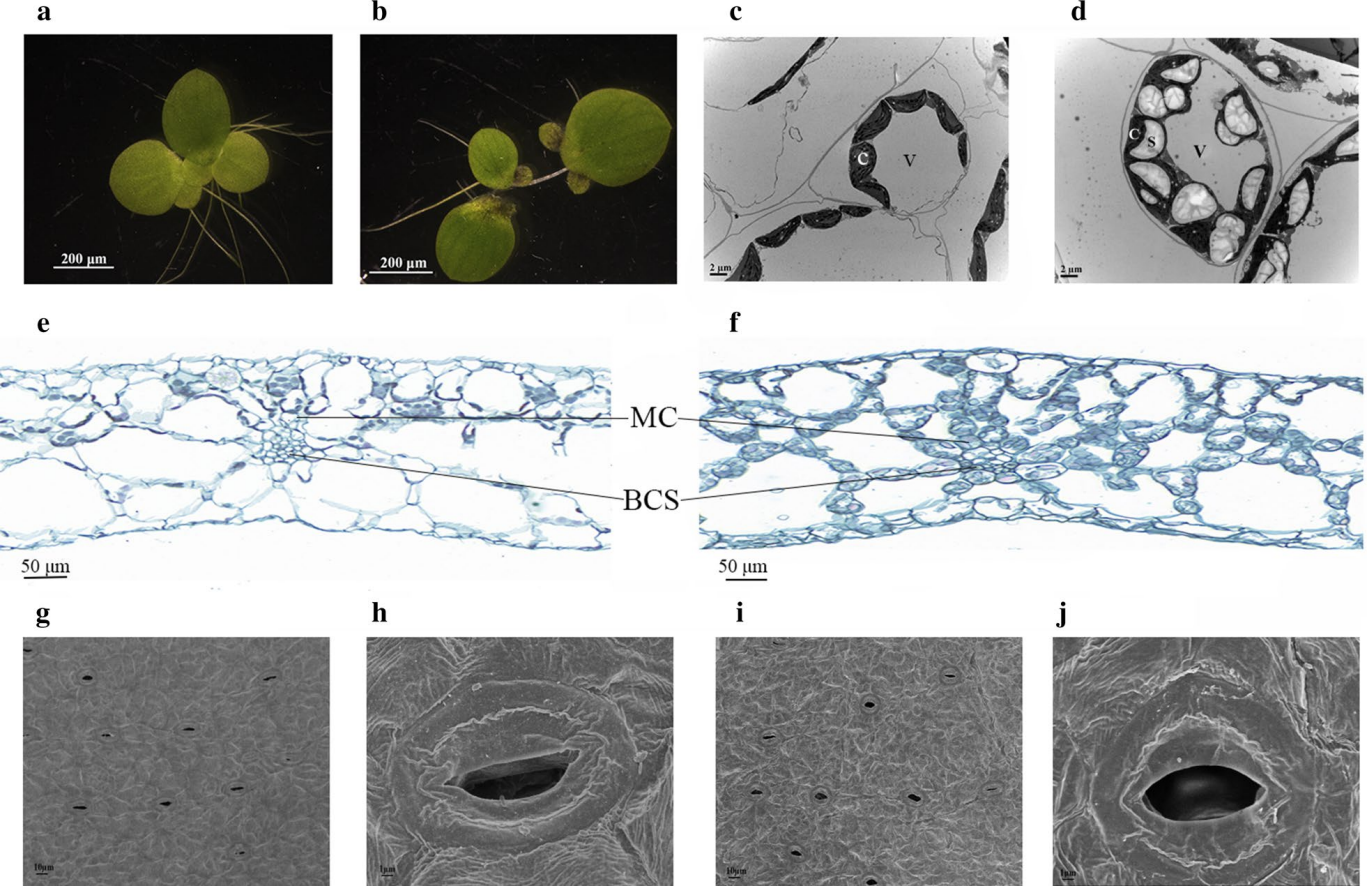
Phenotypic and microscopic observation of *S. polyrrhiza *7498 plants. Phenotypic and microscopic observation were conducted on the 8th day of the experiments. Plants cultivated on medium without (**a**, **c**, **e**, **g** and **h**) or with 75 µg/ml MH (**b**, **d**, **f**, **i** and **j**) were collected at about 4 pm in the day time for observation with a dissecting microscope (**a** and **b**), for frond mesophyll cell observation with TEM (**c** and **d**) and frond stomatal observation with SEM (**g** to **j**). Paraffin slices of fronds were stained by Safranin and Fast Green and observed with light microscope (**e** and **f**). MC indicates mesophyll cell and BSC indicates bundle sheath cell

To the naked eye, the MH-treated fronds looked thicker than that of the controls; therefore, fronds were subjected to dissecting microscope observation and the results confirmed this phenotype change (Fig. 4a, b). Then, paraffin sections (transverse sections) of fronds were prepared and observed with the light microscope. As shown in Fig. 4e, f, the size of the mesophyll cells and vascular bundle sheath cells was larger in the MH-treated fronds than that in the controls. Bigger chloroplasts in the mesophyll cells of MH-treated fronds were also observed in transmission electron microscope (TEM) samples (Fig. 4c, d). The upper surface of the fronds was also observed by scanning electronic microscope (SEM), and the results showed that the opening of stomata in the MH-treated fronds was larger than that of the control (Fig. 4g–j).

### Transcriptomics response to MH treatment

To investigate the transcriptional response of *S. polyrrhiza *7498 plants to MH treatment, RNA-Seq analysis was conducted using RNA samples isolated from fronds treated with 75 µg/mL MH for 8 days, by which fronds grown on medium without supplementation of MH were used as the control.

The KEGG Pathway functional enrichment of differentially expressed genes (DEGs) indicates that MAPK pathway, endocytosis and pathways related to carbon metabolism are mostly affected by MH treatment (Additional file 2: Figure S2). Expression analysis of genes involved in developmental processes by GO classification shows that 39 genes involved in the developmental progress (including many transcriptional factors, some of them being the growth-regulating factors, such as Spo003981, Spo012722 and Spo013751) are down-regulated (Additional file 3: Table S1).

As MH treatment inhibited growth and simultaneously promoted starch accumulation (Figs. 1a, b, 2a, b, d, f), we then mainly focused on photosynthesis, starch biosynthesis and processes related to phytohormone IAA and ABA.

**Fig. 5:**
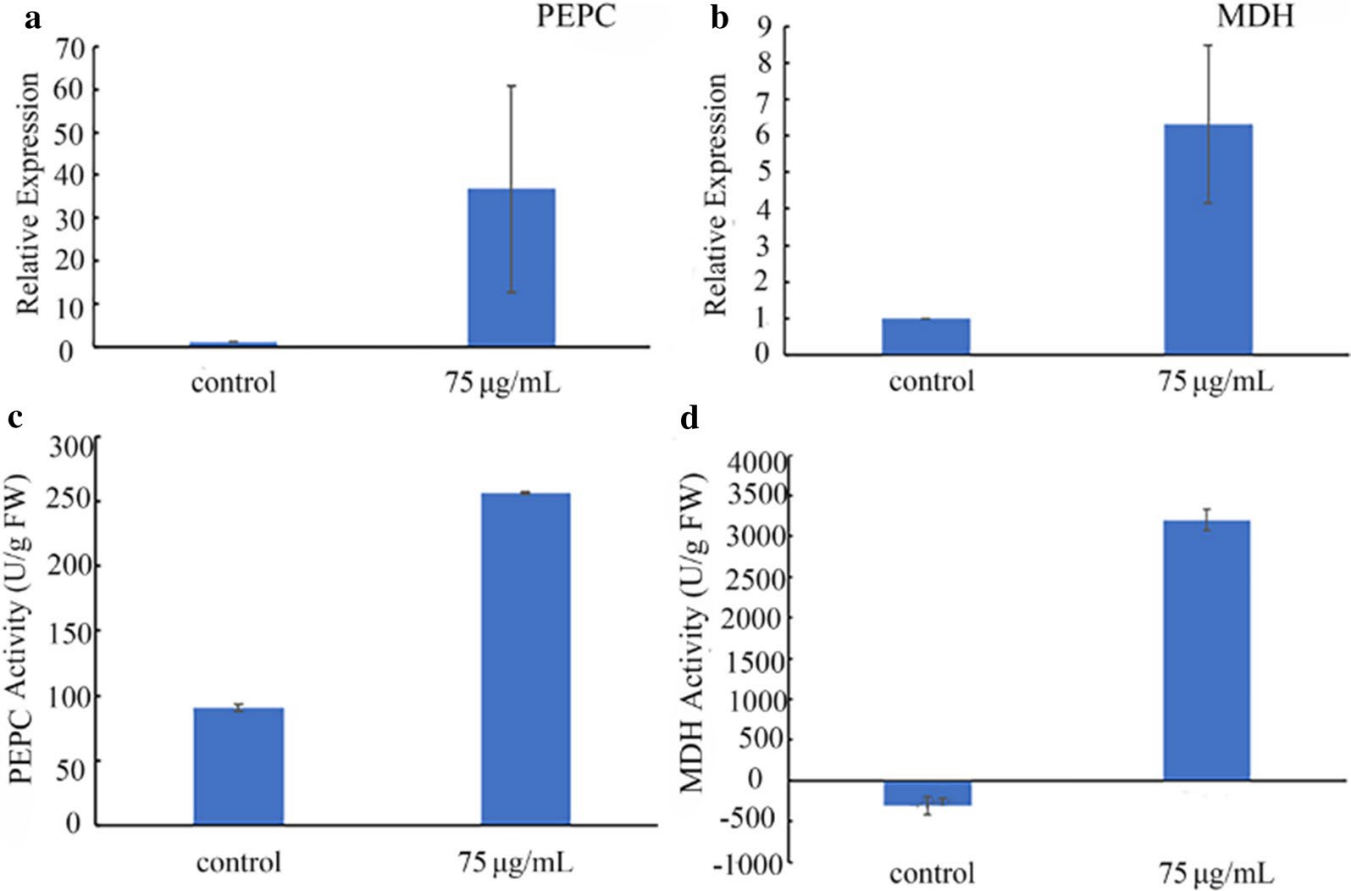
Analysis of gene expression (**a** and **b**) and enzyme activity (**c** and **d**) of the C4 pathway in *S. polyrrhiza* 7498 by qRT-PCR. Plants were cultivated on the medium supplemented with or without 75 µg/mL MH and sampled on the 8th day of cultivation

Photosynthesis is divided into two phases: the light-dependent reactions and the carbon fixation. By light-dependent reactions, ATP and NADPH are generated, which are then used for carbon fixation. As shown in Additional file 4: Table S2, the expression of genes related to light reactions was not significantly affected by MH treatment, except the gene encoding thioredoxin reductase. However, carbon fixation pathways are significantly affected by 75 µg/mL MH treatment. While the expression of some genes involved in Calvin cycle was increased, some other genes involved in the cycle decreased their expression after MH treatment (Table 1). The MH up-regulated genes include those encoding phosphoglycerate kinase [EC:2.7.2.3], glyceraldehyde 3-phosphate dehydrogenase [EC:1.2.1.12], triosephosphate isomerase (TIM) [EC:5.3.1.1], ribose 5-phosphate isomerase A [EC:5.3.1.6] and one of the two ribulose-phosphate 3-epimerase [EC:5.1.3.1]. The MH down-regulated genes include those encoding ribulose-bisphosphate carboxylase small chain [EC:4.1.1.39], glyceraldehyde-3-phosphate dehydrogenase (NADP^+^) (phosphorylating) [EC:1.2.1.13], fructose-1,6-bisphosphatase I [EC:3.1.3.11], transketolase [EC:2.2.1.1], the other ribulose-phosphate 3-epimerase [EC:5.1.3.1] and phosphoribulokinase [EC:2.7.1.19]. Besides, it was found, surprisingly, that the expression of some marker genes of C4 pathway was also significantly up-regulated by MH treatment (Table 1). Duckweed plants are classified as C3 plants [18]. There is no report demonstrating that duckweed has C4 pathway. To confirm our finding, we performed qRT-PCR. The results showed that the expression of the two marker genes encoding PEPC (phosphoenolpyruvate carboxylase) and MDH (NADP-malate dehydrogenase), respectively, were all up-regulated significantly by MH treatment (Fig. 5a, b). We also analyzed the enzyme activity of PEPC and MDH in the plant tissues of *S. polyrrhiza *7498, which showed also a significant increase after MH treatment (Fig. 5c, d).

**Table 1:** Expression analysis of genes involved in carbon fixation

Gene ID	Encoding enzyme	Control (FPKM)	MH (FPKM)	log2
Calvin cycle up-regulated				
Spo018324	Phosphoglycerate kinase [EC:2.7.2.3]	270.05	1145.74	2.09
Spo008868	Glyceraldehyde 3-phosphate dehydrogenase [EC:1.2.1.12]	560.72	7162.6	3.68
Spo009781	Triosephosphate isomerase (TIM) [EC:5.3.1.1]	622.12	1452.24	1.23
Spo013802	Ribose 5-phosphate isomerase A [EC:5.3.1.6]	31.02	186.69	2.59
Spo013803	Ribose 5-phosphate isomerase A [EC:5.3.1.6]	64.84	153.62	1.25
Spo010531	Ribulose-phosphate 3-epimerase [EC:5.1.3.1]	9.83	23.47	1.26
Calvin cycle down-regulated				
Spo005613	Ribulose-bisphosphate carboxylase small chain [EC:4.1.1.39]	66,706.15	12,760.87	− 2.37
Spo007627	Ribulose-bisphosphate carboxylase small chain [EC:4.1.1.39]	1791.85	47.64	− 5.22
Spo001888	Glyceraldehyde-3-phosphate dehydrogenase (NADP^+^) (phosphorylating) [EC:1.2.1.13]	3102.79	638.01	− 2.26
Spo002696	Fructose-1,6-bisphosphatase I [EC:3.1.3.11]	173.34	40.12	− 2.1
Spo003791	Transketolase [EC:2.2.1.1]	573.47	171.25	− 1.73
Spo005573	Ribulose-phosphate 3-epimerase [EC:5.1.3.1]	898.65	386.91	− 1.21
Spo013568	Phosphoribulokinase [EC:2.7.1.19]	1232.06	189.38	− 2.69
C4 pathway up-regulated				
Spo007324	Phosphoenolpyruvate carboxylase [EC:4.1.1.31]	77.16	160.4	1.06
Spo014243	Malate dehydrogenase [EC:1.1.1.37]	525.71	1729.25	1.72
Spo000853	pyruvate, orthophosphate dikinase [EC:2.7.9.1]	86.3	204.32	1.25
Spo007300	Aspartate aminotransferase, mitochondrial [EC:2.6.1.1]	8.56	24.17	1.5
Spo001577	Alanine transaminase [EC:2.6.1.2]	79.38	986.71	3.64
Spo014756	Malate dehydrogenase (oxaloacetate-decarboxylating)(NADP+)[EC:1.1.1.40] /NADP-dependent malic enzyme isoform X1	71.18	140.96	1
Spo018489	Phosphoenolpyruvate carboxykinase (ATP) [EC:4.1.1.49]	162.96	2563.48	3.97
Spo005444	Phosphoenolpyruvate carboxykinase (ATP) [EC:4.1.1.49]	1.05	204.43	7.58
C4 pathway down-regulated				
Spo005743	Phosphoenolpyruvate carboxylase [EC:4.1.1.31]	304.59	137.98	− 1.14

**Fig. 6:**
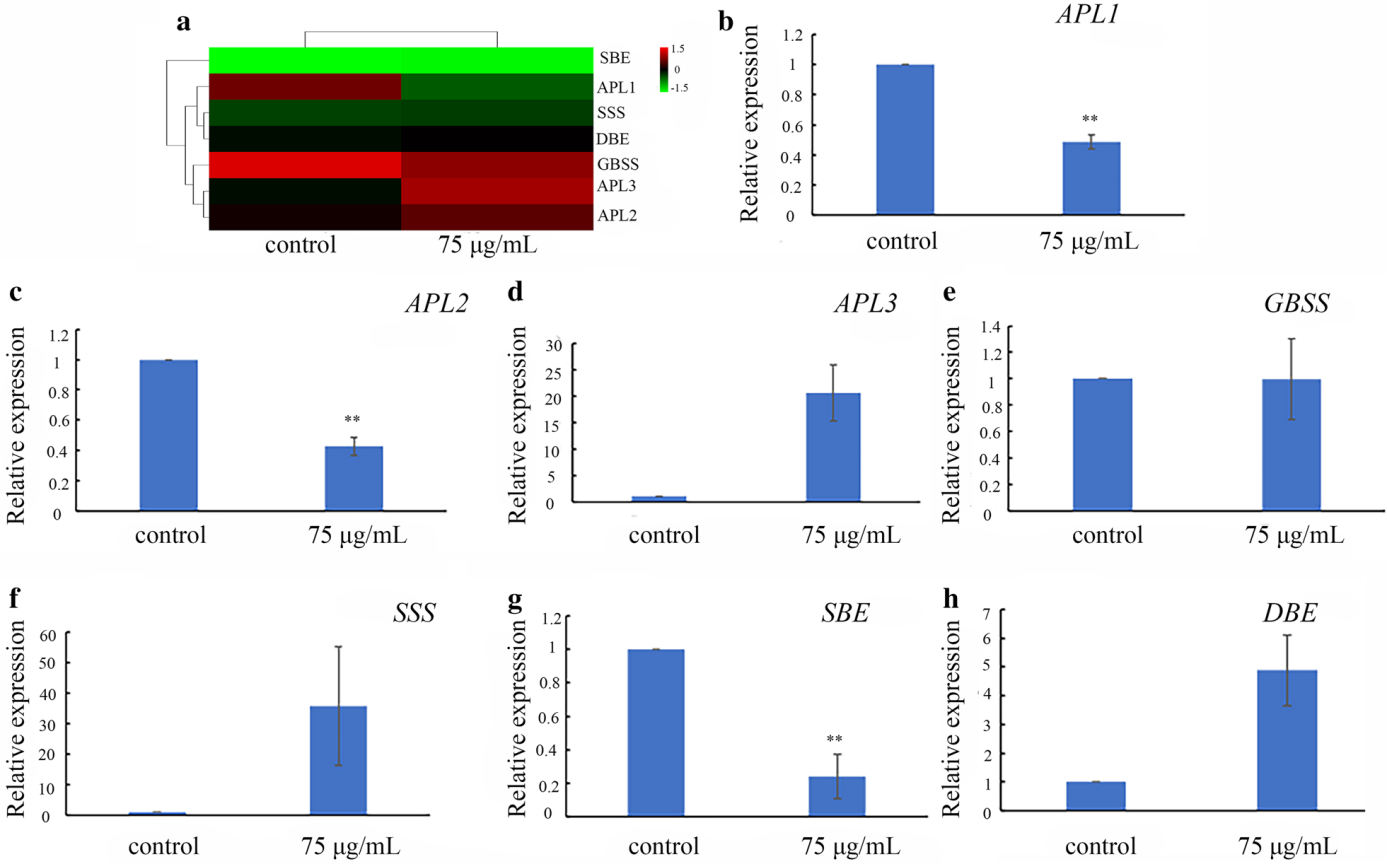
Expression of genes in starch biosynthetic pathway in *S. polyrrhiza* 7498. **a** Expression heatmap, **b**–**h**. qRT-PCR results. **indicates statistically significance (*P* < 0.01). Each point in **b**–**h** indicates mean ± SE of 3 biological replicates. Plants were cultivated in the medium supplemented with or without 75 µg/mL MH and sampled on the 8th day of cultivation

Our RNA-seq data showed that the expression of some transcripts encoding key enzymes involved in the starch biosynthesis pathway could be affected by MH treatment (Fig. 6a). Therefore, we further detected the expression of these genes by qRT-PCR, the results of which confirmed the tendency of the expression of these genes. In detail, MH treatment up-regulated the expression of *APL3* encoding ADP-Glc pyrophosphorylase, *GBSS* encoding granule bound starch synthase, *SSS* encoding soluble starch synthase and *DBE1* encoding starch debranching enzyme*,* but down-regulated the expression of *APL1*, *APL2* and *SBE1* encoding starch branching enzymes (Fig. 6b–h).

Besides, as shown in gene expression heatmaps of KEGG Pathway functional enrichment (Additional file 5: Figure S3, the genes information in the heatmap A, B, C, D and E was listed in the Additional files 10–14, respectively), MH treatment up-regulated the expression of many genes involved in carbon metabolism and lipid degradation.

Auxin is known to regulate many aspects of plant growth and development, and MH is a type of auxin inhibitor. The RNA-Seq results show that the expression of 11 Auxin-related genes was down-regulated and that of 4 genes was up-regulated (Table 2). Analysis of KEGG Orthology shows that these down-regulated DEGs include those encoding the auxin response protein or auxin-induced protein. The phytohormone ABA is well known to be involved in many aspects of plant growth and development, and it has been reported to participate in the regulation of starch biosynthesis [19]. Analysis of DEGs shows that the expression of 9 genes responsible for ABA transport and ABA signaling was up-regulated, and that the expression of 2 and 3 genes involved in ABA regulation was down- or up-regulated, respectively (Table 3).

**Table 2:** Expression analysis of genes involved in auxin response reactions

Gene ID	Control (FPKM)	MH (FPKM)	log2	KEGG Orthology	NR
Down-regulated genes					
Spo004720	18.49	2.18	− 3.05	a,b	Auxin-induced protein 22D-like
Spo001311	274.65	115.53	− 1.24	a,b	Auxin-responsive protein IAA30
Spo001312	84.20	10.32	− 3.02	a,b	Auxin-induced protein 22D-like
Spo004721	117.60	50.00	− 1.23	a,b	Auxin-responsive protein IAA30-like
Spo006931	106.42	20.05	− 2.40	a,b	Auxin-responsive protein IAA27
Spo014766	136.15	23.19	− 2.54	a,b	Auxin-responsive protein IAA27
Spo003507	14.46	4.13	− 1.79	a,c	Auxin-induced protein 6B
Spo015652	23.27	3.38	− 2.75	a,c	Auxin-induced protein
Spo017385	11.19	2.15	− 2.33	a,c	Auxin-responsive protein SAUR32
Spo001587	76.16	9.63	− 2.96	a,c	Auxin-responsive protein SAUR76
Spo005085	50.96	15.02	− 1.75	a,d	GH3 auxin-responsive promoter
Up-regulated genes					
Spo009550	15.69	71.30	2.19	a,e	Auxin transporter-like protein 1 isoform X1
Spo006624	30.71	90.05	1.56	a,b	Auxin-responsive protein IAA1-like
Spo003774	39.74	115.09	1.54	a,c	Auxin-responsive protein SAUR36-like
Spo004635	24.32	205.49	3.08	a,c	Auxin-responsive protein SAUR32-like

^a^Ko04075//Plant hormone signal transduction

^b^K14484//auxin-responsive protein IAA

^c^K14488//SAUR family protein

^d^K14487//auxin responsive GH3 gene family

^e^K13946//auxin influx carrier (AUX1/LAX family)

**Table 3:** Up-regulated DEGs related to ABA signal regulation and DEGs related to ABA response

Gene ID	Control (FPKM)	MH (FPKM)	Log2	NR	
Spo009130	9.40	28.40	1.60	ABC transporter G family member 25	
Spo005834	2.51	7.95	1.66	ABC transporter G family member 10-like	
Spo006246	7.28	20.06	1.46	Protein C2-DOMAIN ABA-RELATED 11 isoform X4	
Spo014819	1.11	14.75	3.70	Protein phosphatase 2C 51 isoform X1	
Spo006751	9.98	28.74	1.52	Protein phosphatase 2C-like domain	
Spo003121	16.56	46.34	1.49	Protein phosphatase 2C 75 isoform X1	
Spo016764	1.84	12.99	2.81	Protein ABSCISIC ACID-INSENSITIVE 5	
Spo000560	3.72	17.53	2.23	ABSCISIC ACID-INSENSITIVE 5-like protein 2	
Spo002610	5.13	11.07	1.11	Hypothetical protein GW17_00034962	
Spo005580	6.33	79.89	3.66	Probable WRKY transcription factor 31	
Spo018210	4.09	44.76	3.44	Probable WRKY transcription factor 50	
Spo002165	15.48	142.78	3.20	Probable WRKY transcription factor 75 isoform X1	
Spo008355	16.43	132.67	3.01	WRKY transcription factor 8	
Spo009313	2.73	16.76	2.60	Probable WRKY transcription factor 48 isoform X2	
Spo006729	21.39	87.56	2.04	Probable WRKY transcription factor 72 isoform X1	
Spo017960	11.31	46.73	2.04	bZIP transcription factor 11-like	
Spo000513	2.03	23.44	3.50	NAC domain-containing protein 68	
Spo000516	26.24	53.12	1.02	NAC domain-containing protein 100	
Spo000068	4.18	13.41	1.68	Transcription factor MYB77-like	
Spo000524	7.11	22.47	1.66	Transcription factor CSA isoform X1	
Spo016388	241.54	69.76	− 1.78	Large subunit ribosomal protein L6	
Spo000564	15.17	4.36	− 1.78	Centromeric-specific histone H3 variant	
Spo006992	22.36	508.03	4.50	PREDICTED:late embryogenesis abundant protein Lea14-A	
Spo012374	34.44	285.02	3.05	Alpha-dioxygenase	
Spo018790	28.48	612.86	4.42	Alpha-dioxygenase	

## Discussion

### Mechanisms underlying growth inhibition by MH

It has been reported that MH, as an inhibitor of auxin, inhibited plant vegetative growth [20–22], which is confirmed in our present research with *S. polyrrhiza* (Fig. 1). The inhibition effect of MH on vegetative growth is very similar to the reported delay of sprouting in onions [21] and yam tubers [20, 22].

Our RNA-Seq analysis for the auxin signal transduction showed that 11 genes expression of auxin response protein or auxin-induced protein was down-regulated (Table 2), which all play a pivotal role in auxin signaling. Among these, the decrease in the expression of genes of Aux/IAA family that encode short-lived nuclear proteins could be related to growth inhibition by MH treatment. MH is an inhibitor of auxin. It has been reported that the expression of genes of the Aux/IAA family are mostly rapidly induced by auxin [23], and mediate the responses of auxin-regulated gene expression [24]. It has also been demonstrated that auxin-mediated transcriptional regulation is exclusively dependent on the functions of Aux/IAA [25]. We also found that IAA22-like, IAA27 and IAA30 (IAA30-like) were all down-regulated in *S. polyrrhiza *7498 after MH treatment (Table 2). Decreasing expression level of these IAA genes may be resulted in the growth inhibition in *S. polyrrhiza *7498*.* Additionally, four genes encoding proteins belonging to the SAURs family were detected to be MH-down-regulated in *S. polyrrhiza* 7498. SAURs are primary auxin response genes involved in the auxin signaling pathway. Their expression could be induced within 2–5 min by active auxin. The regulation of SAURs may occur at the transcriptional, post-transcriptional and protein levels [26, 27]. Inhibition of growth by MH resulted in the decrease in frond propagation (Fig. 1a, b), the production of daughter fronds. It has been reported that the high content of IAA in buds during active growth stage might take the responsibility to the up-regulation of most CsSAURs in *Camellia sinensis* [28]. Accordingly, MH inhibited the growth and propagation of daughter fronds at least partly by down-regulating the expression of SAUR in *S. polyrrhiza *7498. The expression of one *GH3* (Gretchen Hagen 3) gene was also down-regulated by MH treatment. The *GH3* gene family contains important auxin response genes that help maintaining hormonal homeostasis by conjugating excess indole-3-acetic acid (IAA), salicylic acid (SA), and jasmonic acid (JA) to amino acids via hormone- and stress-related signaling pathways [29].

In addition, the expression of some key genes of ABA signaling pathway and ABA response was up-regulated after MH treatment in *S. polyrrhiza* (Table 3). ABA is well known for its inhibition of the plant growth. When ABA treatment was used to induce turion formation (inhibition of growth) in *S. polyrrhiza*, the expression of many genes regulated by ABA, such as large subunit ribosomal protein L6, centromeric-specific histone H3, late embryogenesis abundant protein LEA were affected [16, 30]. The changes of the expression of these genes were similar in fronds after MH treatment (Table 3), suggesting that ABA signaling also plays a role in MH inhibition of vegetative growth.

MH treatment could also affect the expression of genes related to other phytohormone (Additional file 6: Figure S4), and therefore, the involvement of the signaling of those phytohormone should be considered and investigated in the future.

### Mechanisms underlying the starch accumulation after treatment of MH

Higher starch content in tissues of plants, such as cotton, tobacco and *Abelmoschus esculentus* were found after MH treatment [15, 31, 32], which is in accordance with our results (Fig. 2).

The synthesis of starch is inseparable from photosynthesis. The comparison of chlorophyll content and PSII fluorescence parameters suggests that MH does not affect the chlorophyll synthesis and PSII activity in *S. polyrrhiza*, which is in agreement with the results of the report from Koske and Svec [33].

After MH treatment, the fronds of *S. polyrrhiza* looked much thicker than the control, which is also similar to the reports from leaves of bean, sunflower, cotton and *Croft Easter Lilies* following MH application [34, 35]. Besides, we observed larger opening of the stomata in the MH-treated fronds, indicating that the MH-treated fronds have favorable structural features for higher photosynthetic capability. Intriguingly, larger vacuole was observed in *S. polyrrhiza* fronds, being similarly to the characteristics found in *Bienertia cycloptera*, a kind of single-celled C4 species [36, 37]. The large vacuole could separate the inner and outer cytoplasm, and is thought to be the diffusive barrier that slows CO_2_ leakage out of the core cytoplasm in single-celled C4 plant [37]. In addition, increased vein density was reported as a factor for efficient photosynthesis [38]. And the veins were reported colorless in C3 and dark green in C4 [39, 40]. After MH treatment, the veins of the frond also turned a little bit darker, and the expression of genes for C4 pathway increased significantly. Therefore, functioning of C4 pathway in MH-treated fronds could be possible. This is not surprising, as some studies have revealed that C3 plant could exhibit C4 pathway in different organ or different stage of development or under stress [41], and the polyphyletic evolution of the C4 pathway suggested that the transition from C3 to C4 was relatively simple [42]. Increase or activation of C4 pathway could contribute to an increase in carbon fixation. ABA signaling may be involved in the induction of the C4 pathway. In this concern, large quantities of accumulation of key enzymes of the C4 pathway were observed at the appropriate sites of the cells in the C3 plant *Eleocharis vivipara* after 5 µM ABA treatment [43]. However, the Kranz-leaf anatomy was not found in the MH-treated fronds of *S. polyrrhiza *7498.

**Fig. 7:**
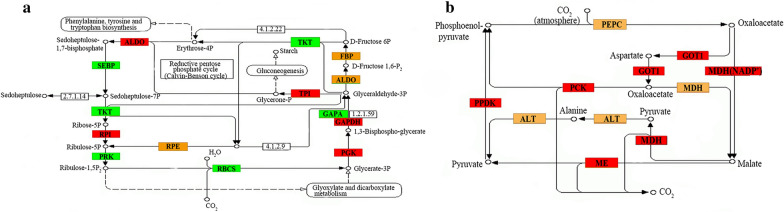
Calvin (left) and C4-Dicarboxylic acid cycle (right) in *S. polyrrhiza* 7498 treated by MH for 8 day. Expression variations of some related transcripts are displayed in carbon fixation pathway. Red boxes indicate the up-regulated enzymes in response to MH, green for down-regulated, orange means the enzyme has more transcripts, one or more up-regulated or down-regulated. And white means this enzyme was not found in this study. TKT: transketolase [EC:2.2.1.1], SEBP: sedoheptulose-bisphosphatase [EC:3.1.3.37], PGK: phosphoglycerate kinase [EC:2.7.2.3], GAPDH: glyceraldehyde 3-phosphate dehydrogenase [EC:1.2.1.12], TPI(TIM): triosephosphate isomerase [EC:5.3.1.1], ALDO: fructose-bisphosphate aldolase, class I [EC:4.1.2.13], FBP: fructose-1,6-bisphosphatase I [EC:3.1.3.11], RPI: ribose 5-phosphate isomerase A [EC:5.3.1.6], RPE: ribulose-phosphate 3-epimerase [EC:5.1.3.1]. RBCS: ribulose-bisphosphatecarboxylase small chain [EC:4.1.1.39], GAPA: glyceraldehyde-3-phosphate dehydrogenase (NADP +) (phosphorylating) [EC:1.2.1.13], RPK: phosphoribulokinase [EC:2.7.1.19]. xfp(xpk): xylulose-5-phosphate/fructose-6-phosphate phosphoketolase [EC:4.1.2.9], [4.1.2.22], SHPK: sedoheptulokinase [EC:2.7.1.14], PEPC: phosphoenolpyruvate carboxylase [EC:4.1.1.31], MDH: malate dehydrogenase [EC:1.1.1.37], PPDK: pyruvate, orthophosphate dikinase [EC:2.7.9.1], GOT1: aspartate aminotransferase, cytoplasmic [EC:2.6.1.1], ALT: alanine transaminase [EC:2.6.1.2], phosphoenolpyruvate carboxykinase (ATP) [EC:4.1.1.49], ME(maeB): malate dehydrogenase (oxaloacetate-decarboxylating)(NADP+) [EC:1.1.1.40], [EC:1.1.1.39]: malate dehydrogenase (decarboxylating), [EC:1.1.1.82]:  malate dehydrogenase (NADP+)

Calvin cycle is responsible for the biosynthesis of sugars from photosynthetic carbon, and therefore, increased expression of the Calvin cycle genes (Table 1) after MH treatment is very understandable. Based on our finding, we propose that MH treatment can not only promote Calvin cycle, but also induce and/or strengthen the operating of the C4 photosynthetic pathway, probably by modifying the ABA signaling in *S. polyrrhiza* 7498, as shown in Fig. 7.

As energy substances, sugars can be used to support plant growth, or converted to starch for storage when growth is inhibited, especially when the photosynthesis is in full function (Figs. 1, 2 and 3; Table 1). Therefore, large amount of starch accumulated in plant tissues after MH treatment required the increase in the activity of many enzymes of starch biosynthesis, which might trigger the elevation of the expression of their encoding genes, such as *APL3*, *SSS* and *DBE* (Fig. 6).

ABA has also been reported to be involved in regulating starch biosynthesis in duckweeds [44], and the starch of fronds was highly accumulated in *S. polyrrhiza*, after ABA treatment for 8 days [6]. The expression of starch biosynthetic gene was found to be closely related with ABA signaling transduction [45, 46]. The expression pattern of *APS1, APL1* and *APL3* in the MH-treated fronds was found to be similar with the results of 10 µM ABA-induced starch accumulation in turions of *S. polyrrhiza *7498 [30, 46]. Therefore, ABA signaling could play an important role during the induction of starch accumulation of fronds after MH treatment. In support of this view, we found that the expression of many key genes of ABA signaling, including several protein phosphatase 2C, protein ABSCISIC ACID-INSENSITIVE 5 and ABA-responsive element binding factor, were up-regulated by MH in *S. polyrrhiza* 7498 (Table 3). Besides, MH and ABA modified similarly the expression of genes related to carbon, glycolysis gluconeogenesis and glycerolipid metabolism (Additional file 5: Figure S3 A–C), as well as the alpha-dioxygenase (Table 3) [30]. Alpha-dioxygenase is a key enzyme responsible for the degradation of lipids, which probably allocate carbon to starch rather than fatty acids.

Previous studies have suggested that there are several kinds of transcription factors that are involved in regulating starch biosynthesis and accumulation, including NAC, WRKY, bZIP, et al. [47–50]. We also found that many genes encoding NAC domain-containing protein and WRKY were up-regulated by MH treatment in *S. polyrrhiza* 7498 (Tables 2 and 3), suggesting that they probably play an important regulating role in the starch synthetic pathway.

## Conclusion

75 µg/mL MH can effectively prevent vegetative growth, and at the same time induce starch accumulation in *S. polyrrhiza *7498. MH could inhibit the frond multiplication in *S. polyrrhiza* by down-regulating the expression of key genes involved in auxin signal transduction and up-regulating the expression of key genes involved in ABA signal transduction. MH could promote the expressions of some genes participating in carbon fixation and starch accumulation, probably through the modification of ABA signaling and regulation of some transcription factors.

This study sheds light on the mechanism(s) of MH on inhibiting vegetative reproduction and inducing starch accumulation in *S. polyrrhiza* 7498, which is closely related with ABA signaling. And the research provides a selective way for increasing duckweed starch accumulation as biofuel source in bioenergy production. The regulated genes by MH treatment would provide good candidates for improving the starch content by genetic engineering in duckweed.

## Materials and methods

### Duckweed strain and treatment

*S. polyrrhiza* (L.) Schleid. 7498 were grown aseptically on Datko medium as described by Wang and Kandeler [51] under long-day conditions (16-h light and 8-h dark) with a light intensity of ∼ 45 µmol m^−2^ s^−1^ at the plant level. The temperature of the culture room was kept at 22 ± 2 °C. *S. polyrrhiza* plants were transferred to fresh medium every 15 days, to minimize the effect of nutrient shortage. For experiments on the effect of MH, six whole *S. polyrrhiza* plants were transferred to medium with different concentration of MH. The plants were cultivated in the same long-day light period condition and temperature, with a light intensity of 100 µmol·m^−2^ s^−1^ at the plant level. MH was from Cool Seoul Bio, Anhui, China.

### Analysis of fronds numbers and weight

For the counting of fronds, fronds were counted with or without MH treatment. Both bigger and smaller fronds were counted and analyzed. For the measurements of fresh weight and dry weight, duckweed plants treated with different concentrations of MH were harvested at different time points. Plants were collected by filtering to separate them from liquid medium, and free water was removed with paper towels. The fresh weight (FW) was measured immediately after which the plants were dried at 85 °C in aluminum foil and weighed every 30 min until the values remained stable, which was recorded as the dry weight (DW).

### Measurements of photosynthetic parameters

Chlorophyll was extracted with 95% ethanol. After centrifugation, the supernatant was collected and used to measure chlorophyll content according to the method of Wintermans and De Mots [52]. Chlorophyll fluorescence parameters were measured with Mini-PAM-II (WALZ, Germany). The value Fv/Fm and Y(II) of photosystem II was measured according to WALZ protocol. Rate of photosynthetic oxygen evolution was measured with Chlorolab 2 (Hansatech, England), according to Hansatech manual.

### Measurements of starch content and enzyme activity

Duckweed plants were sampled after designed time at about 4:00 p.m. in the day time, about 0.1 g fresh weight samples were ground in liquid nitrogen with a mortar and pestle. The starch was then extracted and measured using the starch extraction and measurement kit according to the manufacturer’s protocol (BC0700, Solarbio Biological &Technology Co. Ltd., Beijing, China). For the analysis of PEPC and MDH enzyme activity, we carried out according to the procotol BC2190 and BC1040, respectively (Solarbio Biological &Technology Co. Ltd., Beijing, China). The determination of the starch content and enzyme activity was done in Thermo Multiskan GO (Thermo, USA).

### Observation of fronds morphology and ultrastructure

For observation of the morphology and ultrastructure of frond, the duckweeds were sampled at about 4:00 p.m. in the day time, similar with the starch extracting time after 8 days of cultivation with or without the presence of MH. For the I_2_-KI staining, the duckweed was completely decolorized with 95% ethanol, then stained with I_2_-KI solution for 20 min. Frond morphological and I_2_-KI staining results were observed with dissecting microscope with Leica M165 FC (Germany). Fronds were also prepared in paraffin slices to observe and compare the tissue structure by optical microscopy after staining with Safranin and Fast Green according to the method described by Li [53]. SEM and TEM were prepared and observed by Hangzhou Yanqu Information & Technology Co. Ltd.

For SEM samples, plant tissues were collected and fixed overnight with 2.5% glutaraldehyde solution at 4 °C. The fixed solution was poured out, rinsing the sample with 0.1 M, pH7.0 phosphoric acid buffer for three times, each time for 15 min. The samples were fixed with 1% osmium solution for 1–2 h. Osmium acid waste liquid was carefully taken out, rinsing the sample with 0.1 M, pH7.0 phosphoric acid buffer for three times, each time for 15 min. The samples were dehydrated with ethanol solution of gradient concentration (including 30%, 50%, 70%, 80%, 90% and 95%). Each concentration was treated for 15 min, and then treated with 100% ethanol twice for 20 min. The mixture of ethanol and isoamyl acetate (v/v = 1/1) was used to treat the sample for 30 min; then isoamyl acetate was used to treat the sample for 1 h or overnight. The treated samples were observed with Hitachi SU8010 (Japan) after critical point drying and coating.

For the preparation of TEM samples. The former several steps were same with the SEM samples preparation until treating with 100% ethanol twice for 20 min and finally treated with pure acetone for 20 min. The mixture of embedment and acetone (v/ v = 1/1) was used to treat the sample for 1 h, then the mixture of embedment and acetone (v/v = 3/1) was used to treat the sample for 3 h. The sample treated with pure embedding agent was buried overnight; the embedded sample was embedded and heated at 70 °C overnight, then sliced in Leica EM uc7 ultra-thin microtome, and 70–90 nm sections were obtained. The sections were stained with lead citrate solution and 50% alcohol saturated solution of uranyl acetate for 5–10 min respectively, and then observed with HITACHI H-7650 (Japan).

### RNA-Seq methods and sequence analysis

As described in method, duckweed strain and treatment, there are 3 flasks repeat cultures for control and MH treatment samples prepared for the RNA-Seq, respectively. Whole plants were ground in liquid nitrogen after 8 days culturing, and total RNA was extracted using RNeasy® Plant Mini Kit (Qiagen) according to the manufacturer’s protocol. Integrity of the RNA was confirmed by gel electrophoresis and total RNA was then submitted to Beijing Genomics Institute (BGI-Shenzhen), Shenzhen, China (http://www.genomics.cn) for RNA-Seq, library construction and sequencing. The sample RNA was sequenced with the DNBSEQ platform, the number of detected genes and average sample-to-genome alignment rate was shown in Additional file 7: Table S3. Reference Genome Alignment Analysis software was HISAT (Hierarchical Indexing for spiced Alignment of Transcripts (version: V2.1.0). Clean reads were compared to reference gene sequences using BOWTIE2 (version: V2.2.5), and then RSEM was used to calculate gene and transcript expression levels. The analysis of the filtered Reads quality shown in Additional file 8: Table S4. Differential expressed genes between samples were identified, and clustering analysis and functional annotations were done according to the annotation information from *S. polyrrhiza* kindly shared by Professor Wenqing Wang [54]. The significance of the differential expression of genes was defined by the bioinformatics service of BGI according to the combination of the absolute value of log2-Ratio ≥ 1 and false discovery rate (FDR) ≤ 0.001. KOG functional classification, Gene Ontology (GO) and pathway annotation and enrichment analyses were based on the Gene Ontology Database (http://www.geneontology.org/) and KEGG pathway database (http://www.genome.jp/kegg/), respectively. The software Cluster and Java Treeview were used for hierarchical cluster analysis of gene expression patterns [55, 56].

### RNA extraction and qRT-PCR

RNA was isolated from *Spirodela polyrrhiza* 7498 which were treated with 75 μmol/L MH for different days, using an Eastep Super Total RNA Extraction Kit (Promega, shanghai, China). After reverse transcription was performed, the cDNAs for *INO1* (Inositol-3-phosphate synthase, used as a control), *APL1*, *APL2*, *APL3*, *GBSS*, *SSS, SBE, DBE*, *PEPC* and *MDH* were amplified using qRT-PCR with specific primers (in Additional file 9: Table S5). qRT-PCR was performed on an iCycler Thermal Cycler (Bio-Rad iQ5, Hercules, CA, USA) using TB Green Premix Ex TaqII (Code No. RR420A:TB Green® Premix Ex Taq™, Dalian Takara, Dalian, China) according to the manufacturer’s protocol. The reaction mixture was heated to 95 °C for 30 s followed by 40 PCR cycles at 95 °C for 5 s, 58 °C for 30 s and 72 °C for 30 s. All primer pair efficiencies were between 95 and 105%, and the individual efficiency values were considered in the calculation of normalized relative expression. The difference in the relative expression levels of *APL1*, *APL2*, *APL3*, *GBSS*, *SSS, SBE, DBE*, *PEPC* and *MDH* was calculated using the 2^−ΔΔCT^ method after the data were normalized to *INO1*. All values are shown as the mean ± standard error of the mean using at least three biological replicates.

## Supplementary Information

**Additional file 1: Figure S1.** Comparison of root length in *S. polyrrhiza 7498*.

**Additional file 2: Figure S2.** Pathway functional enrichment of DEGs.

**Additional file 3: Table S1.** Expression analysis of genes involved in developmental progress by GO classification.

**Additional file 4: Table S2**. Expression analysis of genes up-regulated in light reactions.

**Additional file 5: Figure S3.** Main pathway expression heatmap of DEGs classified by KEGG.

**Additional file 6: Figure S4.** Plant hormone signal transduction induced by MH in *S. polyrrhiza*.

**Additional file 7: Table S3.** Number of detected genes and alignment rate of RNA-Seq analysis.

**Additional file 8: Table S4.** Summary of sequence read alignments to reference genome.

**Additional file 9: Table S5.** Primers used in the qRT-PCR.

**Additional file 10: Table S6.** Gene information of carbon metabolism pathway expression heatmap.

**Additional file 11: Table S7.** Gene information of glycolysis gluconeogenesis.

**Additional file 12: Table S8.** Gene information of glycerolipid metabolism pathway expression heatmap.

**Additional file 13: Table S9.** Gene information of MAPK pathway expression.

**Additional file 14: Table S10.** Gene information of carbon fixation pathway.

## Data Availability

All data generated or analyzed during this study are included in this published article.

## Abbreviations

MH: Maleic hydrazide
ABA: Abscisic acid
6-BA: 6-Benzylamino-purine
AIP: 2-Aminoindan-2-phosphonic acid
DEGs: Differentially expressed genes
AUX1/LAX: Auxin influx carrier
Aux/IAA: Auxin/indole-3-acetic acid
SAUR: Small auxin up-regulated RNA
ATP: Adenosine triphosphate
NADPH: Reduced form of nicotinamide adenine dinucleotide phosphate
TIM: Triosephosphate isomerase
PEPC: Phosphoenolpyruvate carboxylase
MDH: NADP-malate dehydrogenase
AGPase: ADP-Glc pyrophosphorylase
GH3: Gretchen Hagen 3
FW: Fresh weight
DW: Dry weight
FDR: False discovery rate
GO: Gene Ontology
*INO1*: Inositol-3-phosphate synthase
TEM: Transmission electron microscope
SEM: Scanning electronic microscope
