# Supplementary material for: Molecular mechanism underlying the effect of maleic hydrazide treatment on starch accumulation in S. polyrrhiza 7498 fronds
Source: Biotechnol Biofuels. 2021 Apr 19;14:99. doi: 10.1186/s13068-021-01932-y (PMC8056677; doi:10.1186/s13068-021-01932-y)
Supplement: Supplementary file 1 — Additional file 1: Figure S1. Comparison of root length in S. polyrrhiza 7498. [file 13068_2021_1932_MOESM1_ESM.docx]

**Additional file**

**Additional file 1 Figure S1**

**
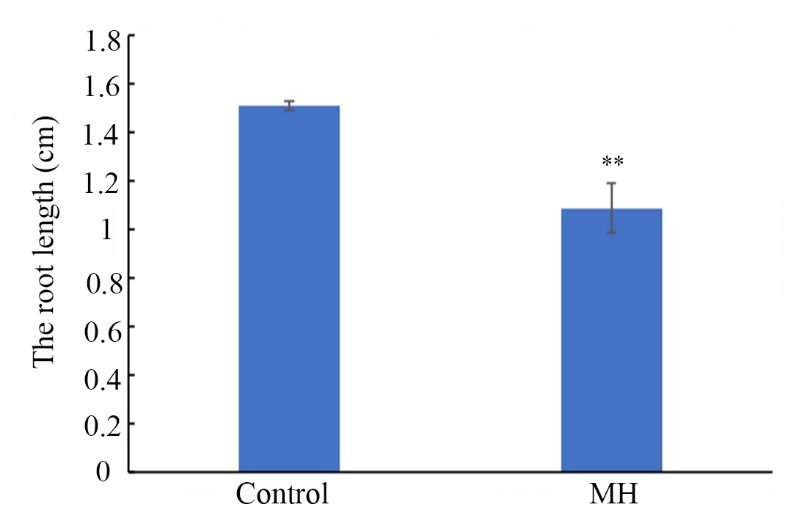
**

Figure S1 Comparation of root length in *S. polyrrhiza 7498*

Six groups of duckweed plants (total 24 fronds) were inoculated, the root length was compared on the 8^th^ day of experiment with 0 µg/mL (control) and 75 µg/mL MH treatment, respectively.  **indicates statistically significance (P<0.01). Each point in the figure mean ± SE of 3 biological replicates.
