# Supplementary material for: Molecular mechanism underlying the effect of maleic hydrazide treatment on starch accumulation in S. polyrrhiza 7498 fronds
Source: Biotechnol Biofuels. 2021 Apr 19;14:99. doi: 10.1186/s13068-021-01932-y (PMC8056677; doi:10.1186/s13068-021-01932-y)
Supplement: Supplementary file 2 — Additional file 2: Figure S2. Pathway functional enrichment of DEGs. [file 13068_2021_1932_MOESM2_ESM.docx]

**Additional file 2 Figure S2**

**
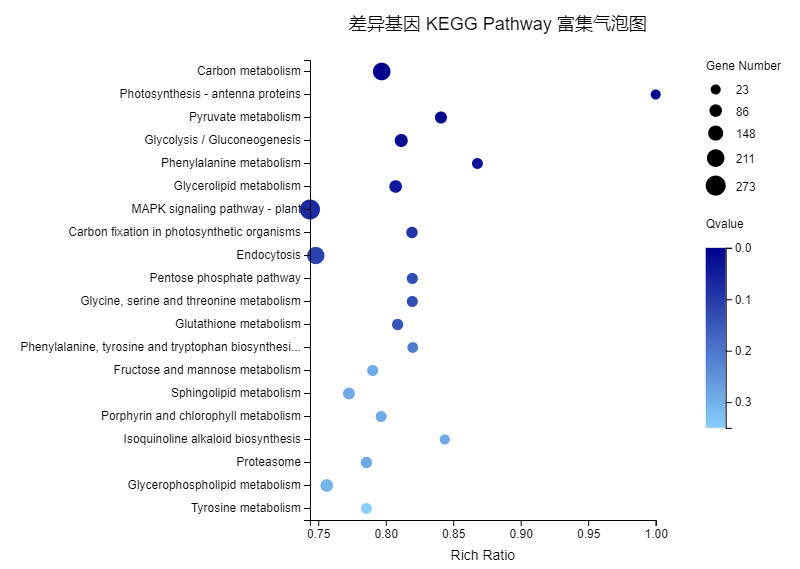
**

Figure S2 Pathway functional enrichment of DEGs

X axis represents enrichment factor. Y axis represents pathway name. The color indicates the q-value (high: white, low: blue), a lower q-value indicates more significant enrichment. Point size indicates DEG number. Rich Factor refers to the value of enrichment factor, which is the quotient of foreground value (the number of DEGs) and background value (total gene amount). The larger the value, the more significant enrichment.
