# Supplementary material for: Molecular mechanism underlying the effect of maleic hydrazide treatment on starch accumulation in S. polyrrhiza 7498 fronds
Source: Biotechnol Biofuels. 2021 Apr 19;14:99. doi: 10.1186/s13068-021-01932-y (PMC8056677; doi:10.1186/s13068-021-01932-y)
Supplement: Supplementary file 3 — Additional file 3: Table S1. Expression analysis of genes involved in developmental progress by GO classification. [file 13068_2021_1932_MOESM3_ESM.docx]

**Additional file 3. Table S1**

Table S1. Expression analysis of genes involved in developmental progress by GO classification

| Gene ID | Control | MH（FPKM） | log2 | TFs | NR |
| --- | --- | --- | --- | --- | --- |
|  | (FPKM) |  |  |  |  |
| Spo017424 | 15.2 | 7.03 | -1.03 |  | AT-hook motif nuclear-localized protein 22-like |
| Spo006378 | 13.91 | 6.68 | -1.09 |  | BTB/POZ domain-containing protein NPY1 |
| Spo017425 | 19.23 | 8.04 | -1.13 |  | AT-hook motif nuclear-localized protein 26 |
| Spo014516 | 12.65 | 5.64 | -1.13 |  | probable protein NAP1 |
| Spo000743 | 15.2 | 6.74 | -1.15 |  | PREDICTED: kinesin-like protein KIN-13A |
| Spo011021 | 13.73 | 5.94 | -1.18 |  | protein PIR isoform X2 |
| Spo015225 | 18.45 | 7.64 | -1.25 |  | hypothetical protein C4D60_Mb01t24520 |
| Spo011552 | 22.87 | 8.56 | -1.41 |  | serine--tRNA ligase, chloroplastic/mitochondrial isoform X1 |
| Spo011379 | 36.65 | 13.36 | -1.45 |  | PREDICTED: B-box zinc finger protein 20 |
| Spo011040 | 173.15 | 61.87 | -1.52 |  | HXXXD-type acyl-transferase family protein |
| Spo014374 | 28.98 | 9.8 | -1.6 |  | homeobox-leucine zipper protein HDG2 isoform X4 |
| Spo011651 | 15.94 | 5.9 | -1.65 |  | hypothetical protein GW17_00001556 |
| Spo011838 | 15.52 | 4.46 | -1.74 |  | GRF1-interacting factor 1 |
| Spo001595 | 40.52 | 11.56 | -1.76 |  | tyrosine--tRNA ligase, chloroplastic/mitochondrial |
| Spo016388 | 238.4 | 69.76 | -1.78 |  | 50S ribosomal protein L6, chloroplastic |
| Spo007510 | 21.63 | 5.29 | -1.99 |  | PREDICTED: fasciclin-like arabinogalactan protein 4 |
| Spo013782 | 19.46 | 5.05 | -2 |  | PREDICTED: CEN-like protein 1 |
| Spo013282 | 17.12 | 4.32 | -2.02 |  | uncharacterized protein LOC103716503 |
| Spo017649 | 9.25 | 2.16 | -2.1 |  | PREDICTED: uncharacterized protein LOC104604527 isoform X2 |
| Spo003981 | 14.29 | 3.18 | -2.13 |  | growth-regulating factor 4 |
| Spo004532 | 19.15 | 2.12 | -3.08 |  | ABC transporter B family member 19 |
| Spo014011 | 27.33 | 2.08 | -3.4 |  | organic cation/carnitine transporter 1 |
| Spo001998 | 10.69 | 0.7 | -3.95 |  | PREDICTED: protein SIEVE ELEMENT OCCLUSION B |
| Spo011578 | 46.08 | 2.13 | -4.34 |  | PREDICTED: sphinganine C4-monooxygenase 2-like |
| Spo004215 | 22.88 | 10.99 | -1.09 | C2C2-YABBY | PREDICTED: putative axial regulator YABBY 2 isoform X2 |
| Spo006776 | 28.63 | 9.9 | -1.39 | C2C2-GATA | GATA transcription factor 21-like |
| Spo001163 | 15.47 | 5.18 | -1.52 | MYB | transcription factor MYB61 |
| Spo004301 | 29.5 | 9.2 | -1.68 | C2C2-GATA | GATA transcription factor 2-like |
| Spo011026 | 64.64 | 20.28 | -1.73 | C2C2-GATA | hypothetical protein BAE44_0020044 |
| Spo002623 | 24.26 | 6.87 | -1.78 | MYB | myb-related protein 308-like |
| Spo003254 | 11.96 | 2.94 | -2 | MYB | LOW QUALITY PROTEIN: transcription factor MYB41-like |
| Spo006858 | 83.27 | 19.61 | -2.07 | MYB | transcription factor RAX1-like |
| Spo012722 | 25.84 | 4.91 | -2.42 | GRF | growth-regulating factor 4-like isoform X2 |
| Spo003768 | 22.21 | 3.88 | -2.44 | C2C2-YABBY | protein YABBY 4-like |
| Spo013751 | 9.46 | 1.31 | -2.84 | GRF | growth-regulating factor 10-like |
| Spo017775 | 13.52 | 1.59 | -2.97 | C2C2-GATA | GATA transcription factor 12 |
| Spo014712 | 16.16 | 1.84 | -3.1 | C2C2-GATA | putative GATA transcription factor 22 |
| Spo003723 | 43.42 | 3.4 | -3.53 | C2C2-GATA | GATA transcription factor 2-like |
| Spo001498 | 33.92 | 2.83 | -3.55 | GRF | WRC |
