# Supplementary material for: Molecular mechanism underlying the effect of maleic hydrazide treatment on starch accumulation in S. polyrrhiza 7498 fronds
Source: Biotechnol Biofuels. 2021 Apr 19;14:99. doi: 10.1186/s13068-021-01932-y (PMC8056677; doi:10.1186/s13068-021-01932-y)
Supplement: Supplementary file 4 — Additional file 4: Table S2. Expression analysis of genes up-regulated in light reactions. [file 13068_2021_1932_MOESM4_ESM.docx]

**Additional file 4. Table S2**

Table S2. Expression analysis of genes up-regulated in light reactions

| Gene ID | Encoding enzyme | | Control | MH | log2 | Pvalue |
| --- | --- | --- | --- | --- | --- | --- |
| Spo016615 | thioredoxin reductase | 2.4 | | 96.98 | 5.37 | 6.5E-144 |
| Spo008720 | ferredoxin--NADP+ reductase | | 64.48 | 108.29 | 0.75 | 2.55E-12 |
| Spo015623 | ferredoxin-2 | | 13.36 | 24.65 | 0.89 | 1.86E-06 |
| Spo015718 | H^+^-transporting ATPase | | 313.7 | 569.14 | 0.86 | 1.02E-23 |
