# Supplementary material for: Molecular mechanism underlying the effect of maleic hydrazide treatment on starch accumulation in S. polyrrhiza 7498 fronds
Source: Biotechnol Biofuels. 2021 Apr 19;14:99. doi: 10.1186/s13068-021-01932-y (PMC8056677; doi:10.1186/s13068-021-01932-y)
Supplement: Supplementary file 5 — Additional file 5: Figure S3. Main pathway expression heatmap of DEGs classified by KEGG. [file 13068_2021_1932_MOESM5_ESM.docx]

**Additional file 5 Sup. Figure 3**

**A**

**
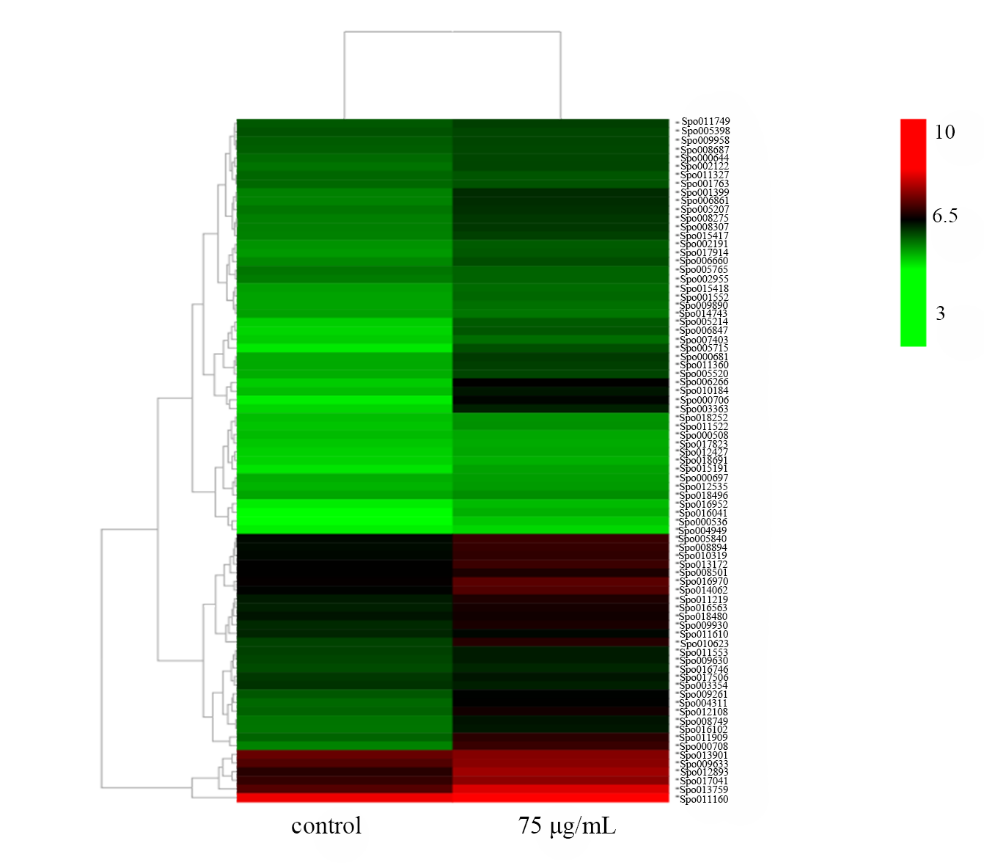
**

**B**

**
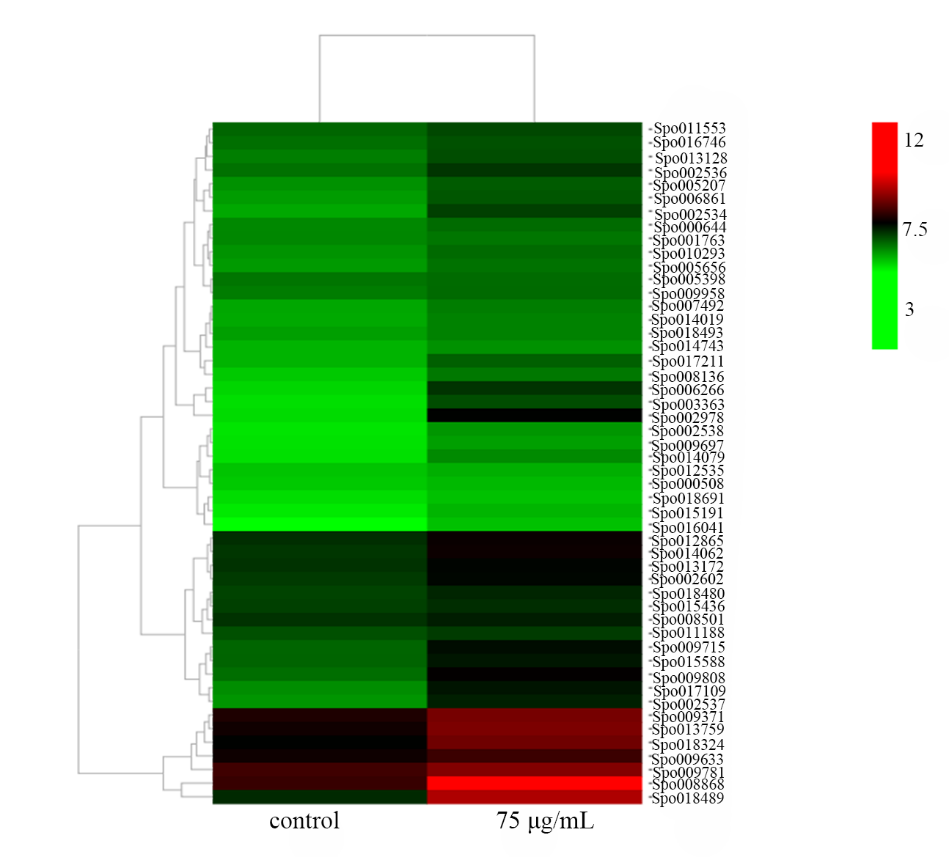
**

**C**

**
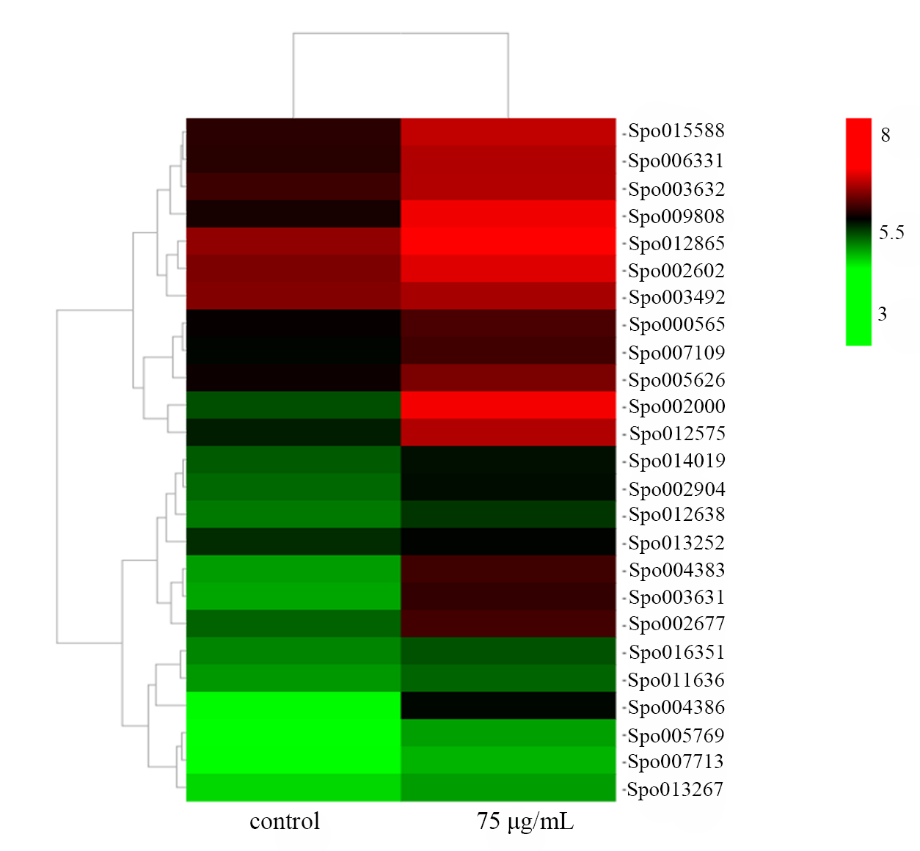
**

**D**

**
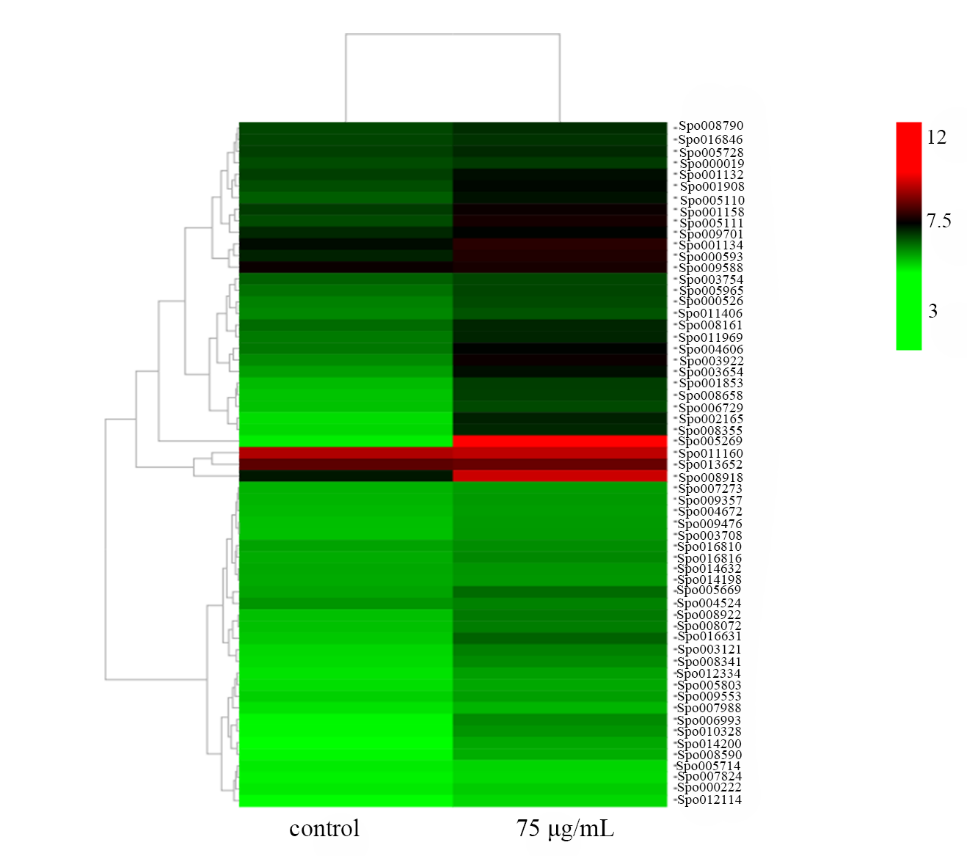
**

**E**

**
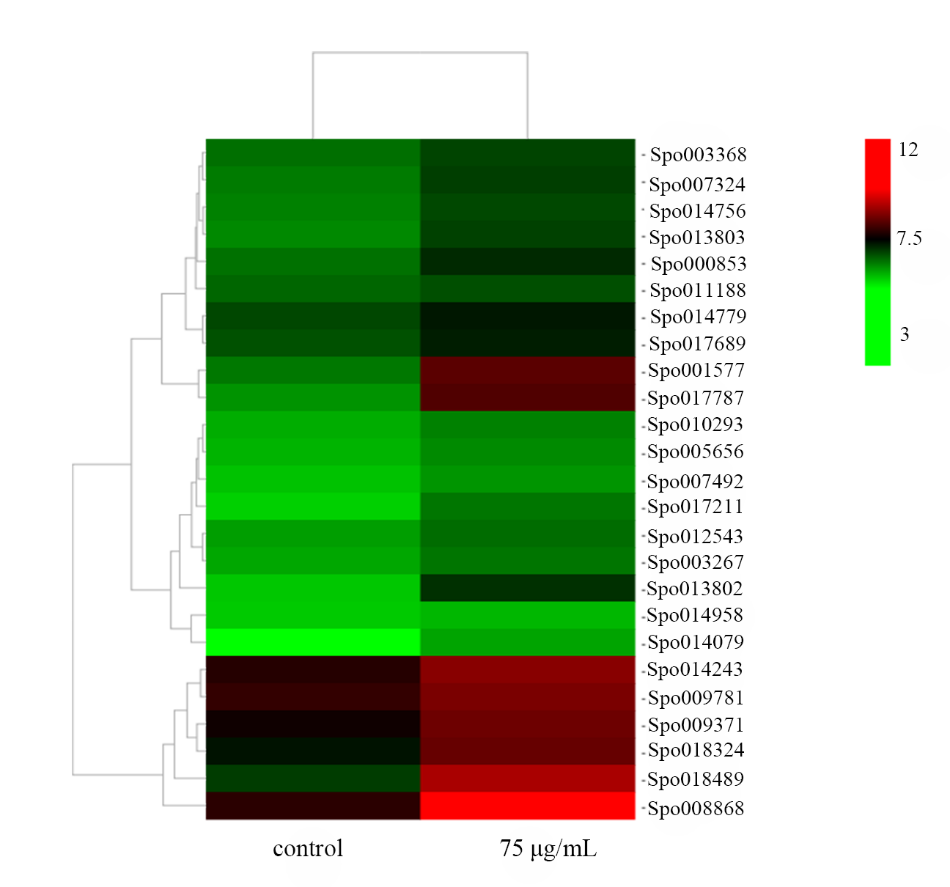
**

Fig.3 Main pathway expression heatmap of DEGs classified by KEGG

A. Carbon metabolism; B. Glycolysis gluconeogenesis; C. Glycerolipid metabolism; D. MAPK; E. Carbon fixation. Information of these genes in the heatmap was list in the Additional file 10-14, respectively.
