# Supplementary material for: Molecular mechanism underlying the effect of maleic hydrazide treatment on starch accumulation in S. polyrrhiza 7498 fronds
Source: Biotechnol Biofuels. 2021 Apr 19;14:99. doi: 10.1186/s13068-021-01932-y (PMC8056677; doi:10.1186/s13068-021-01932-y)
Supplement: Supplementary file 6 — Additional file 6: Figure S4. Plant hormone signal transduction induced by MH in S. polyrrhiza. [file 13068_2021_1932_MOESM6_ESM.docx]

**Additional file 6 Figure S4.**

**
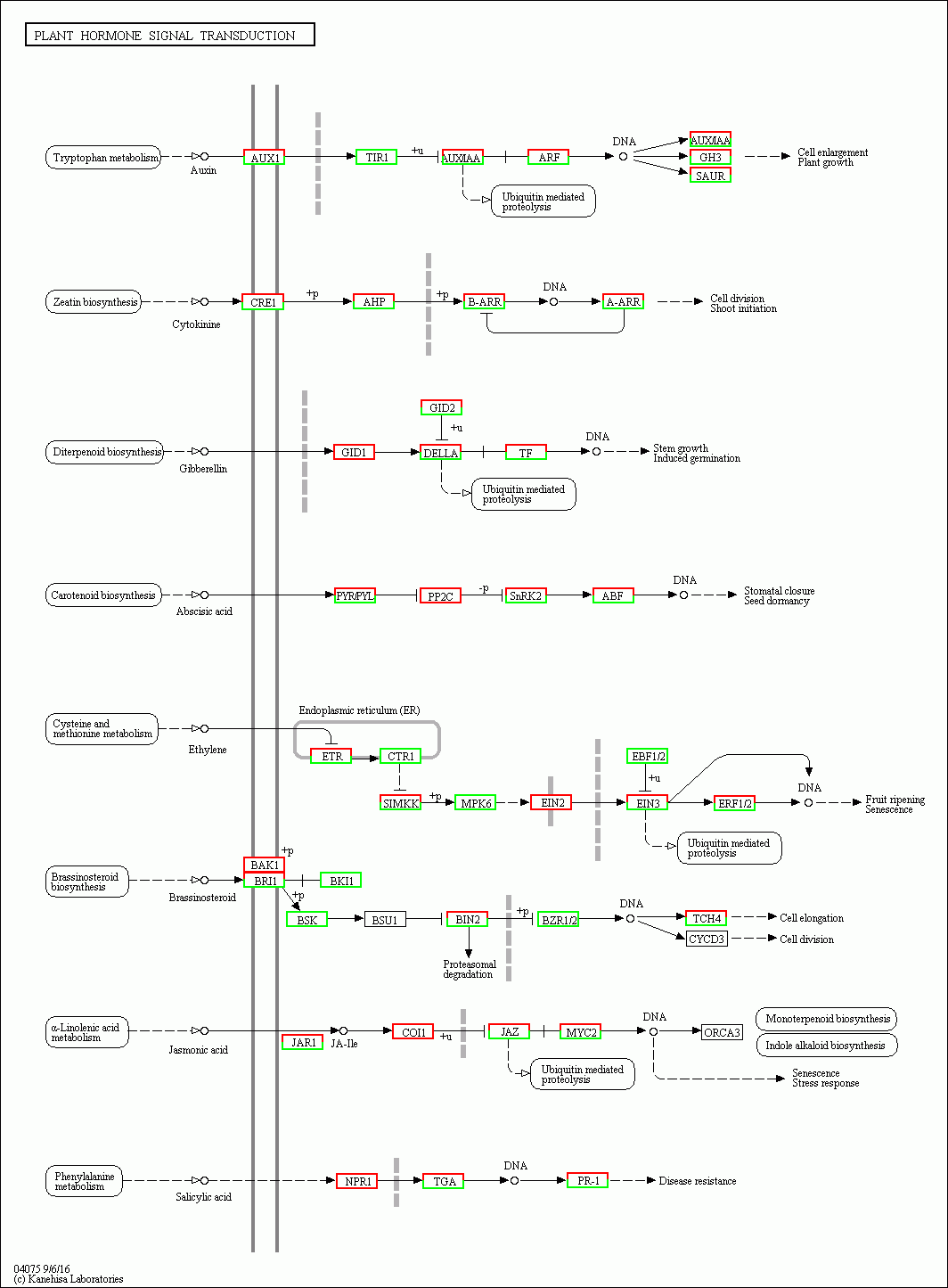
**

Figure S4 Plant hormone signal transduction induced by MH in *S. polyrrhiza*
