# Supplementary material for: Molecular mechanism underlying the effect of maleic hydrazide treatment on starch accumulation in S. polyrrhiza 7498 fronds
Source: Biotechnol Biofuels. 2021 Apr 19;14:99. doi: 10.1186/s13068-021-01932-y (PMC8056677; doi:10.1186/s13068-021-01932-y)
Supplement: Supplementary file 7 — Additional file 7: Table S3. Number of detected genes and alignment rate of RNA-Seq analysis. [file 13068_2021_1932_MOESM7_ESM.docx]

**Additional file 7 Table S3.**

Table S3. Number of detected genes and alignment rate of RNA-Seq analysis

| Total detected Gene | Average alignment rate of sample to genome | Average alignment rate of sample to gene set |
| --- | --- | --- |
| 17642 | 95.09% | 70.56% |
