# Supplementary material for: Molecular mechanism underlying the effect of maleic hydrazide treatment on starch accumulation in S. polyrrhiza 7498 fronds
Source: Biotechnol Biofuels. 2021 Apr 19;14:99. doi: 10.1186/s13068-021-01932-y (PMC8056677; doi:10.1186/s13068-021-01932-y)
Supplement: Supplementary file 8 — Additional file 8: Table S4. Summary of sequence read alignments to reference genome. [file 13068_2021_1932_MOESM8_ESM.docx]

**Additional file** **8 Table S4.**

Table S4. Summary of sequence read alignments to reference genome

| Sample | Total Raw Reads (M) | | Total Clean Reads (M) | Total Clean Bases(Gb) | Clean Reads Q20(%) | Clean Reads Q30(%) | Clean Reads Ratio(%) |
| --- | --- | --- | --- | --- | --- | --- | --- |
| MH_8D_0_1 | 21.29 | 21.29 | | 1.06 | 98.44 | 90.96 | 100 |
| MH_8D_0_2 | 21.26 | | 21.26 | 1.63 | 98.36 | 90.47 | 100 |
| MH_8D_0_3 | 21.36 | | 21.36 | 1.07 | 98.32 | 90.4 | 100 |
| MH_8D_75_1 | 21.21 | | 21.36 | 1.06 | 98.5 | 91.1 | 100 |
| MH_8D_75_2 | 21.2 | | 21.2 | 1.06 | 98.37 | 90.55 | 100 |
| MH_8D_75_3 | 21.38 | | 21.38 | 1.07 | 98.48 | 90.91 | 100 |
