# Supplementary material for: Molecular mechanism underlying the effect of maleic hydrazide treatment on starch accumulation in S. polyrrhiza 7498 fronds
Source: Biotechnol Biofuels. 2021 Apr 19;14:99. doi: 10.1186/s13068-021-01932-y (PMC8056677; doi:10.1186/s13068-021-01932-y)
Supplement: Supplementary file 9 — Additional file 9: Table S5. Primers used in the qRT-PCR. [file 13068_2021_1932_MOESM9_ESM.docx]

**Additional file 9 Sup. Table 5.**

Table 5. Primers used in the qRT-PCR

| Gene | 5′ Primer | 3′ Primer |
| --- | --- | --- |
| *APL1*(Spo000868) | GCACCGTAGAGCATAGCG | TCGTCCTCCGTCTCATAG |
| *APL2*(Spo018468) | ACCGTCGAGCATTCCATC | GCGTTCTTCACCACCACC |
| *APL3*(Spo018109) | GCAGCATCCAGCATTCCA | CGCCTCGGTCTGATAGTTGT |
| *GBSS*(Spo003469) | ATCCAGAACTGCATGGCTCA | TCGCTCCCACCAGAAAGG |
| *SSS*(Spo007204) | TGCGACATACTCTTAATGCCCT | CCACTGGAAGACTTGCTCGTA |
| *SBE*(Spo007736) | TGGACGTGCTTACTTAAACTTC | CTTATCAAAGTTGAAAATACTGCTA |
| *DBE*(Spo001017) | GTAATAAAGGCTGGCATCG | ACACGGTTTCACAGAAGG |
| *PEPC*(Spo007324) | CGGCGTTCAAGCATGTCC | CGTTGAGCGTCGTGGTGTAG |
| *MDH*(Spo014243) | GGGGCTCATCTACTCCTTC | TGGCGTCCATCTTCTTCC |
| *INO1*(Spo001754) | ACTCCTTCCACCCAGTAGCC | TCTCCAGCATCGCCCTCT |
